# Genetic dissection of *Striga hermonthica* (Del.) Benth. resistance via genome-wide association and genomic prediction in tropical maize germplasm

**DOI:** 10.1007/s00122-020-03744-4

**Published:** 2021-01-03

**Authors:** Manje Gowda, Dan Makumbi, Biswanath Das, Christine Nyaga, Titus Kosgei, Jose Crossa, Yoseph Beyene, Osval A. Montesinos-López, Michael S. Olsen, Boddupalli M. Prasanna

**Affiliations:** 1 International Maize and Wheat Improvement Center (CIMMYT), Village Market, P. O. Box 1041, 00621 Nairobi, Kenya; 2 Moi University, P. O. Box 3900-30100, Eldoret, Kenya; 3 International Maize and Wheat Improvement Center (CIMMYT), Apdo, Postal 6-641, 06600 Mexico, D.F Mexico; 4 Facultad de Telemática, Univ. de Colima, 28040 Colima, México

## Abstract

**Key message:**

Genome-wide association revealed that resistance to *Striga hermonthica* is influenced by multiple genomic regions with moderate effects. It is possible to increase genetic gains from selection for Striga resistance using genomic prediction.

**Abstract:**

*Striga hermonthica* (Del.) Benth., commonly known as the purple witchweed or giant witchweed, is a serious problem for maize-dependent smallholder farmers in sub-Saharan Africa. Breeding for *Striga* resistance in maize is complicated due to limited genetic variation, complexity of resistance and challenges with phenotyping. This study was conducted to (i) evaluate a set of diverse tropical maize lines for their responses to *Striga* under artificial infestation in three environments in Kenya; (ii) detect quantitative trait loci associated with *Striga* resistance through genome-wide association study (GWAS); and (iii) evaluate the effectiveness of genomic prediction (GP) of *Striga*-related traits. An association mapping panel of 380 inbred lines was evaluated in three environments under artificial *Striga* infestation in replicated trials and genotyped with 278,810 single-nucleotide polymorphism (SNP) markers. Genotypic and genotype x environment variations were significant for measured traits associated with *Striga* resistance. Heritability estimates were moderate (0.42) to high (0.92) for measured traits. GWAS revealed 57 SNPs significantly associated with *Striga* resistance indicator traits and grain yield (GY) under artificial *Striga* infestation with low to moderate effect. A set of 32 candidate genes physically near the significant SNPs with roles in plant defense against biotic stresses were identified. GP with different cross-validations revealed that prediction of performance of lines in new environments is better than prediction of performance of new lines for all traits. Predictions across environments revealed high accuracy for all the traits, while inclusion of GWAS-detected SNPs led to slight increase in the accuracy. The item-based collaborative filtering approach that incorporates related traits evaluated in different environments to predict GY and *Striga*-related traits outperformed GP for *Striga* resistance indicator traits. The results demonstrated the polygenic nature of resistance to *S. hermonthica*, and that implementation of GP in *Striga* resistance breeding could potentially aid in increasing genetic gain for this important trait.

**Supplementary Information:**

The online version contains supplementary material available at (10.1007/s00122-020-03744-4).

## Introduction

The purple witchweed or giant witchweed, *Striga hermonthica* (Del.) Benth., is the most widespread parasitic weed posing a serious threat to maize production in sub-Saharan Africa (SSA) (Berner et al. 1995; De Groote et al. 2008; Spallek et al. 2013). *Striga hermonthica* (hereafter referred to as *Striga*) lacks its own root system and survives by drawing water and nutrients from host plants like maize for its own growth and has a potent phytotoxic effect (Bebawi and Mutwali 1991). Maize plants infested with *Striga* become chlorotic, produce thin stalks with severe reduction in plant height, biomass and eventually grain yield (Menkir et al. 2012, 2020). *Striga* infestation is severe in areas with poor soil fertility and poorly managed but intensively cultivated farming systems (Ransom 2000). Many farmers experience total maize crop failure due to *Striga* infestation. Farmers who faced 100% yield loss usually move from one affected field to another, and abandoned fields become *Striga* seed banks. It is estimated that *Striga* infestation causes up to US$ 7 billion in crop losses (Berner et al. 1995), affecting the livelihoods of over 100 million people (Badu-Apraku and Akinwale 2011).

*Striga* infestation is extremely difficult to control since the parasite inflicts most of its damage when it is below the ground and emerges after most weeding operations are completed (Odhiambo and Ransom 1995). The emerged parasitic plants act as strong sink for host resources to support their own growth, flowering and seed production (Gurney et al. 1995, 1999). Several *Striga* plants attach to a single maize plant as parasites; thus, their impact on maize biomass and grain yield is often devastating and may lead to 100% yield loss (Ransom et al. 1990; Haussmann et al. 2000; Kim et al. 2002). Several methods have been proposed for *Striga* management, including host plant resistance, cultural, chemical and manual control options (Odhiambo and Ransom 1995; Kim et al. 2002). Integrated *Striga* management is a strategy involving a combination of two control methods, where the methods are used simultaneously to control *Striga* (Kanampiu et al. 2018). However, the use of host plant resistance is considered the most economical, environmentally viable and affordable for smallholder or resource-constrained farmers in SSA. Progress in breeding for native genetic resistance to *Striga* in maize has been reported in several studies (Menkir et al. 2005, 2012; Badu-Apraku and Lum 2007; Badu-Apraku et al. 2016; Menkir and Meseka 2019). Efforts to incorporate herbicide resistance in maize for *Striga* control have also been reported (Kanampiu et al. 2001; Makumbi et al. 2015).

Genome-wide association study (GWAS) enables genetic dissection of complex traits. GWAS offers high mapping resolution and could effectively identify favorable genomic regions associated with the trait of interest (Yu and Buckler 2006). Linkage disequilibrium (LD) decay is rapid in maize due to its extensive genetic diversity (Kump et al. 2011; Guo et al. 2013). Therefore, GWAS needs to be implemented with a large number of high-quality markers to ensure complete coverage of the genome. To date, GWAS has been successfully applied to identify the quantitative trait nucleotide (QTN) or haplotypes conferring resistance to several important diseases of maize, such as gray leaf spot (Shi et al. 2014), Southern corn leaf blight (Kump et al. 2011), Fusarium ear rot (Zila et al. 2013), maize lethal necrosis (Gowda et al. 2015; Nyaga et al. 2019; Sitonik et al. 2019) and sugarcane mosaic virus (Tao et al. 2013; Gustafson et al. 2018). Recently, Adewale et al. (2020) reported first GWAS on *Striga* resistance traits with small set of 132 early maturing inbred lines. On the other hand, in this study, we used GWAS panel with 380 lines for identifying genomic regions controlling resistance to *Striga hermonthica*.

Crop breeders need innovative methods that aid in selection for improvement of complex traits such as *Striga* resistance. Genomic prediction (GP) facilitates prediction of best-performing lines and can potentially accelerate the breeding cycle with optimal resources (Crossa et al. 2017). In a maize breeding program, GP-based selection is comparable to a traditional selection scheme; however, GP produces considerable savings in both time and resources (Combs and Bernardo 2013; Beyene et al. 2015, 2019). In general, results of random cross-validation with genomic best linear unbiased predictor (GBLUP) indicate that GP can significantly increase prediction accuracy for complex traits (Crossa et al. 2017). Further, GBLUP models could be extended to multi-environment settings where G × E effects are modeled to improve the prediction accuracy. Earlier studies on complex traits like grain yield (Burgueño et al. 2012; Jarquín et al. 2014; Juliana et al. 2018) clearly showed that by modeling G × E using both pedigree and markers, prediction accuracy could be increased substantially. Therefore, in this study, we ascertained the potential of GP for a complex trait like *Striga* resistance with and without modeling the G × E effects.

The trait of interest to be predicted could be affected by variability in the correlated traits; for instance, grain yield under artificial *Striga* infestation could be affected by other *Striga* resistance-related traits, disease and other agronomic traits. Therefore, these traits could be considered for inclusion in prediction models. Several statistical models which incorporate multiple traits in GP are available (Montesinos-López et al. 2016) but to fit these factors into models is complex and time-consuming. Recently, a new algorithm called ‘item-based collaborative filtering’ (IBCF) was reported to be competitive with respect to computing time and prediction accuracy (Montesinos-López et al. 2018a). IBCF is popularly used for recommending items/products in electronic commerce websites, where a list of recommended items/products is generated based on customer’s interests. The IBCF uses inputs about a customer’s interests to generate predictions. This algorithm has been implemented in GP and proved to be comparable and sometimes even superior to conventional whole-genome prediction models when the correlation between traits and environments was moderate or high (Montesinos-López et al. 2018b). Recently, the IBCF recommender system was applied for multivariate predictions of traits in maize and wheat breeding (Juliana et al. 2018, 2019; Montesinos-López et al. 2018b).

While several studies have reported the use of GWAS to understand the genetic architecture of some biotic stresses, e.g., (Zila et al. 2013; Ding et al. 2015; Gowda et al. 2015; Mammadov et al. 2015; Kuki et al. 2018; Nyaga et al. 2019; Sitonik et al. 2019) and abiotic stresses in maize, e.g., (Yuan et al. 2019; Ertiro et al. 2020), its application for identification of genomic regions associated with resistance to *Striga* has not been reported. Also, GP has been applied to several traits in maize (Burgueño et al. 2012; Gowda et al. 2015; Gustafson et al. 2018; Sitonik et al. 2019; Yuan et al. 2019) but not for *Striga* resistance. The overall aim of this study was to dissect the genetic basis underlying *Striga* resistance in maize under artificial infestation and to identify targets for knowledge-based improvement of *Striga* resistance in tropical maize germplasm. The specific objectives of the study were to: (i) evaluate the diverse array of 380 tropical maize inbred lines for response to *Striga* under artificial infestation; (ii) detect main-effect QTL and putative candidate genes associated with *Striga* resistance; (iii) assess the utility of GP for *Striga* resistance with different cross-validation methods in the tropical maize panel; and (iv) evaluate multivariate predictions of *Striga* resistance indicator traits using other correlated agronomic traits based on the IBCF approach. We evaluated the IBCF approach for predicting a given target trait (for example, GY and *Striga* resistance indicator traits) by incorporating information from other traits that can affect the target trait(s). In this study, users represent the target trait while items represent other related traits evaluated in different environments, that are expected to have some correlation with the target trait.

## Materials and methods

### Plant materials and field trials

This study used the Improved Maize for African Soils (IMAS) association mapping panel (Gowda et al. 2015) of 380 CIMMYT maize inbred lines. All the inbred lines were developed by International Maize and Wheat Improvement Center (CIMMYT) and International Institute for Tropical Agriculture (IITA) breeding programs for drought, low N, soil acidity (SA) and pest and disease resistance, through conventional breeding methods. The list of the inbred lines, the source germplasm and the method employed for the development of the lines can be found at http://www.data.cimmyt.org. This panel broadly represents tropical and subtropical maize genetic diversity, including germplasm derived from diverse breeding programs at CIMMYT (Wen et al. 2011).

## Field evaluation, experimental design and artificial infestation with *Striga hermonthica*

The 380 inbred lines were evaluated in replicated trials in three environments (environment 1—Kibos2013, environment 2—Alupe2013 and environment 3—Alupe2014) under artificial *Striga* infestation during the long rainy seasons (March–August). Both Kibos [− 0.03861°S, 34.81596°E; 1,193 m above mean sea level (masl); 865 mm mean annual rainfall] and Alupe (0.503725°N, 34.12148°E; 1153 masl; 1400 mm mean annual rainfall) have a bimodal rainfall distribution. *Striga* seeds collected from sorghum fields were stored for few months which helps to break the seed dormancy which was later used for the infestation. Artificial infestation of the *S. hermonthica* trials at Kibos and Alupe was carried out by adding viable *Striga* seeds to each planting hole. Each maize plant was exposed to a minimum of 2000 viable *S. hermonthica* seeds. A standard scoop calibrated to deliver specified amount of germinable *Striga* seeds per hill was used for the artificial infestation (Kim 1991). *Striga* seeds collected earlier from farmers maize and sorghum fields around Kibos station, containing about 25% extraneous material and 25% viability in 10 g of soil/seed mixture, were added to an enlarged planting hole at a depth of 7–10 cm directly below the maize, as per the protocol optimized by Kanampiu et al. (2018) to ensure that each maize plant was exposed to *Striga* at germination. Two maize seeds were placed into the holes infested with sand–*Striga* seeds mixture and then covered with soil. The experimental design used was 5 by 76 simple alpha-lattice with two replications at both locations. Plot size was two rows, with a spacing of 0.75 m and 0.25 m between rows and plants, respectively. Plots were thinned to one plant per hill two weeks after planting to attain a population density of approximately 53,333 plants per hectare. The recommended fertilizer rate was reduced, and application was delayed up to three weeks after planting to induce the production of strigolactones which stimulate good germination of the *Striga* seeds and the attachment of the *Striga* plants to the roots of host plants (Adewale et al. 2020; Badu-Apraku et al. 2020a, b). Weeding was done by hand to remove all weeds from the field except *S. hermonthica*. IMAS panel was also evaluated in Striga-free environments, under *Striga*-free conditions as well as under low N (Ertiro et al. 2020) and under maize lethal necrosis conditions (Sitonik et al. 2019). However, in this study, we focused only on data collected under *Striga* infestation environments.

## Data recording

Grain yield, host plant *Striga* damage syndrome rating and emerged *Striga* plant counts were recorded in the field trials. Host plant *Striga* damage syndrome rating (SDR) was visually rated for each plot at 10 and 12 weeks after planting (WAP) using a scale of 1–9, where 1 to 3 = no visible or mild damage symptoms; 4 to 5 = some leaf blotching, wilting and stunting; 6 to 8 = extensive leaf scorching, noticeable stunting and reduction in plant growth; and 9 = all leaves completely scorched and dead (Kim 1994). The number of emerged *S. hermonthica* plants was recorded at 8, 10 and 12 WAP. The emerged *Striga* plant counts at the three intervals were used to calculate the “area under the *Striga* number progress curve” (AUSNPC), using the formula for “area under the disease progress curve” (AUDPC) (Shaner and Finney 1977; Haussmann et al. 2000).

Other agronomic traits including days to anthesis (AD, days from planting to when 50% of the plants had shed pollen) and days to silking (SD, days from planting to when 50% of the plants had extruded silks) were recorded. Plant height (PH, measured in centimeters as the distance from the base of the plant to the height of the first tassel branch), ear height (EH, measured in centimeters), number of ears per plant (EPP, determined by dividing the total number of ears per plot by the number of plants harvested per plot), husk cover (HC, obtained by dividing the number of ears with poor husk cover by the number or plants harvested per plot), plant aspect (PASP, rated on a scale of 1–5, where 1 = excellent plant type and 5 = poor plant type) and grain moisture at the time of harvest were also recorded. All the ears harvested from each plot were weighed, and representative samples of ears were shelled to determine percentage moisture using a Dickey John moisture meter at all locations. Grain yield (GY, t ha^−1^) was calculated from ear weight and grain moisture, assuming a shelling percentage of 80% and adjusted to 12.5% grain moisture content. These agronomic traits data were collected to understand their correlations and their usefulness in the breeding for *Striga* resistance. Further other traits are also used in multivariate predictions for *Striga*-related traits and GY.

## Phenotypic data analyses

All quantitative genetic parameters were estimated based on the performance of the 380 inbred lines in the IMAS association mapping panel. Residuals for all traits were normally distributed. Individual location analyses were performed, and data from locations with significant genotypic variation and good repeatability were selected for across location analyses. We removed the outliers and did the across location analyses. Analyses of variance within and across environments were undertaken by the restricted maximum likelihood method using the software ASREML-R (Gilmour et al. 2009). The following linear mixed model was used for analysis:

$$Y_{ijko} = \mu + G_{i} + \, E_{j} + \, \left( {{\text{GE}}} \right)_{ij} + \, R\left( E \right)_{kj} + \, B\left( {{\text{R}}.{\text{E}}} \right)_{ojk} + e_{ijko}$$

where *Y*_*ijko*_ is the phenotypic performance of the *i*th genotype at the *j*th environment in the *k*th replication of the *o*th incomplete block, *µ* is an intercept term, *G*_*i*_ is the genetic effect of the *i*th genotype, *E*_*j*_ is the effect of the *j*th environment, *R(E)*_*kj*_ is the effect of the *k*th replication at the *j*th environment, *B(R.E)*_*ojk*_ is the effect of the *o*th incomplete block in the *k*th replication at the *j*th environment and *e*_*ijko*_ is the residual. Environments and replications were treated as fixed effects and the other effects as random. Heritability on an entry-mean basis was estimated from the variance components on a progeny mean basis as described by Hallauer and Miranda (1981): $h^{2} = \, \sigma^{2}_{G} / \, (\sigma^{2}_{G} + \, \sigma^{2}_{{{\text{GXE}}}} /E \, + \, \sigma^{2}_{e} /{\text{ER}}$), where $\sigma^{2}_{G} , \, \sigma^{2}_{{{\text{GXE}},}} \sigma^{2}_{e}$ refer to the genotypic, genotype X environment interaction and error variances, and E and R indicate the number of environments and replications, respectively. Best linear unbiased predictions (BLUPs) and best linear unbiased estimates (BLUEs) were calculated using META-R software (Alvarado et al. 2015) (https://hdl.handle.net/11529/10201). Pairwise Pearson’s correlation coefficients (r) among the traits were calculated using R software version 3.2.5 (https://www.r-project.org/).

## Molecular data analyses

Detailed description of the IMAS panel and their genotyping with genotyping-by-sequencing (GBS) markers was described earlier (Gowda et al. 2015; Ertiro et al. 2020). In brief, DNA of all inbred lines extracted from 2–3 weeks old seedlings was genotyped using GBS (Elshire et al. 2011) at Cornell University, Ithaca, USA, as per the procedure described earlier (Elshire et al. 2011; Glaubitz et al. 2014). SNPs which were polymorphic, having minor allele frequency of > 0.05, with < 5% of missing data, and heterozygosity of < 5%, were reserved for final GWAS analysis. Applying these filtering metrics, 278,810 polymorphic SNPs were retained for GWAS in the IMAS panel.

BLUPs calculated for *Striga* count at 8, 10 and 12 WAP, AUSNPC, SDR and GY at each environment and across environments were used in GWAS. Trait data were corrected for population structure using the general linear model (GLM), as well as population structure and kinship (Q + K) using the mixed linear model (MLM) algorithm (Flint‐Garcia et al. 2005; Yu and Buckler 2006). GWAS and principal component (PC) analysis were performed using TASSEL ver 4.0 (Bradbury et al. 2007). Population structure was corrected by using the first three PCs which explained the maximum variation. For multiple testing correction to determine the significance threshold, instead of 278,810 independent tests, the total number of tests was estimated based on the average extent of LD at *r*^2^ = 0.1 (Cui et al. 2016, 2017). Based on this, significant associations were declared when the *P* values in independent tests were less than 2 × 10^−06^ for *Striga* resistance traits and *P* = 5.6 × 10^–6^ for GY. The total proportion of phenotypic variance explained by the detected QTL was calculated by fitting all significant SNPs simultaneously in a linear model to obtain *R*^2^_adj_. The proportion of the genotypic variance explained by all QTLs was calculated as the ratio of *p*_*G*_ = *R*^2^_adj_/*h*^2^. Candidate genes containing or adjacent to the significant SNPs were obtained from the B73 gene set in Maize GDB (https://www.maizegdb.org/gene_center/gene).

### Multivariate predictions of Striga-related traits and grain yield

We used the IBCF approach for multivariate prediction of GY and *Striga*-associated traits in individual environments using its similarity to other traits measured at different environments. The detailed steps involved in IBCF approach implementation are described in earlier studies (Juliana et al. 2017, 2018; Montesinos-López et al. 2018a). In brief, the basic idea of IBCF algorithm is building a database of users (lines) by preferences for items (trait-environment combination). Then, each column of this matrix ($\left[ {z_{ij} = (y_{ij} - \mu_{j} } \right)\sigma_{j}^{ - 1} ]$) is standardized, where $i$ denotes the users (lines), $j$ denotes the columns (trait-environment combinations), ${\mu }_{j}$ is the mean of column $j$ and ${\sigma }_{j}$ denotes the standard deviation of column$j$. Then, the Pearson correlation between the columns of the resulting standardized matrix (trait-environment combinations) is computed. Next with the following formula (Sarwar et al. 2001; Montesinos-López et al. 2018a), the predictions for the missing phenotypes of line $i$ in item $j$ are computed.

$$\hat{y}_{ij} = \mu_{j} + \sigma_{j} \hat{z}_{ij}$$

where $\hat{z}_{ij} = \frac{{\sum\nolimits_{{j^{\prime } \int {N_{i} \left( j \right)} }} {z_{{ij^{\prime } }} w_{{jj^{\prime } }} } }}{{\sum\nolimits_{{j^{\prime } \int {N_{i} \left( j \right)} }} {w_{{jj^{\prime } }} } }}$ is the predicted scaled phenotype for user (line) $i$ on item (trait-environment) $j.$ $N_{i}$(*j*) denotes the items rated by user (line) $i$ most similar to item *j*, $w_{{jj^{\prime } }}$ is the weight between items $j$ and $j^{\prime }$ and the weights are obtained from an item-to-item similarity matrix built using the Pearson’s correlation, which provides information on how similar an item is to another item. We implemented IBCF to predict each trait from one environment using other traits from remaining environments in the complete set of lines using the ‘R’ package IBCF.MTME (Montesinos-López et al. 2018a).

## Genomic prediction

High similarities have been reported among available GP models (Juliana et al. 2017). In this study, we used the whole-genome regression approach GBLUP which employed the genomic relationship matrix (*G*-matrix) calculated from markers (VanRaden 2008) and has been successfully applied to predict complex traits (Habier et al. 2013; Yang et al. 2017). The GBLUP model was implemented in the ‘*R*’ package BGLR (Pérez and de Los Campos 2014). The models include genomic effect within environment, and multi-environment including environment and genomic main effects and genomic × environment interaction (G × E). First, we provide the model used within each environment, with only the main effects of genotypes (genomic) in the predictor:

1 $$y_{j} = G_{j} + \varepsilon_{j}$$

where $y_{j}$ represents the normal response observed in the $j$-th line with $j = 1,2, \ldots J.$ $G_{j}$ represents the genotype effect of the $j$-th line and is assumed as a random effect distributed as $G = \left( {G_{1} , \ldots , G_{J} } \right)^{T} \sim N\left( {0,G_{1} \sigma_{G}^{2} } \right)$, where $G_{1}$ denotes the genomic relationship matrix calculated as suggested by (VanRaden 2008) and $\sigma_{G}^{2}$ denotes the genomic variance. Finally, ${\varepsilon }_{j}$ is the random error term associated with the $j$-th line distributed $N\left( {0,\sigma_{e}^{2} } \right)$, where $\sigma_{e}^{2}$ denotes the residual variance. Next, the model containing environmental main effects and the genotype $\times$ environment interaction is

2 $$y_{ij} = E_{i} + G_{j} + EG_{ij} + \varepsilon_{ij}$$

where $${y}_{ij}$$ represented the normal response observed in the $$j$$-th line at the $$i$$-th environment, where $$i = 1,2, \ldots ,I;\;j = 1,2, \ldots J.$$ The main effect of environments $$E_{i} \sim$$ $$N\left( {0,I\sigma_{E}^{2} } \right)$$ (where ***I*** is the identity matrix and $$\sigma_{E}^{2}$$ is the variance of environments) $${\text{EG}}_{ij}$$ is the interaction between the genotype effect of the $$j$$-line with the $$i$$-th environment and is also assumed as a random effect distributed as $${\text{GE}} = \left( {{\text{EG}}_{11} , \ldots , {\text{EG}}_{IJ} } \right)^{T} \sim$$$$N\left( {0,G_{1} \otimes I_{I} \sigma_{{{\text{GE}}}}^{2} } \right)$$, where $$\sigma_{{{\text{GE}}}}^{2}$$ denotes the variance of the interaction of G × E. Finally, $$\varepsilon_{ij}$$ is the random error term associated with the $$j$$-th line in the $$i$$-th environment distributed $$N\left( {0,\sigma_{e}^{2} } \right)$$, where $$\sigma_{e}^{2}$$ denotes the residual variance.

## Evaluation of prediction performance

The IBCF was used only for one type of cross-validation (CV) scheme and was found very useful in breeding programs, this cross-validation called incomplete field trials (CV2) was the practical method when some lines were evaluated in some environments but not in others; the goal here was to predict the performance of these lines in environments where they had not been phenotyped (Crossa et al. 2017). However, the GBLUP model in addition to the CV2 cross-validation also evaluated the prediction of new lines (CV1) in an attempt to measure the predictive ability of new lines that had not been phenotyped in any environments, predictive ability between phenotyped and unphenotyped lines were primarily based on genetic similarities as the main source of information, and predicting already observed lines in unobserved environments (CV0; leaving one environment out). Here, the main interest was to predict the performance of lines in potentially new locations. For random cross-validation CV0, CV1 and CV2, the prediction accuracies were calculated by performing random fivefold cross-validation where 20% of the maize lines (testing set) were predicted and 80% were used as training set. For CV1, none of the 20% of the lines in the testing set was observed in any of the environments, whereas for CV2 (in both the GBLUP and IBCF), the 20% of the lines in the testing set were observed in some environments but not in the others. The prediction accuracy was computed as the correlations between the observed and predicted values. Additionally, one more CV was carried out where accuracies were predicted within IMAS association mapping panel by using BLUEs across environments. The GP was carried out with and without inclusion of significant markers detected in GWAS analyses for the respective traits. For each trait, the sampling of training and validation sets was repeated 100 times.

## Results

**Fig. 1:**
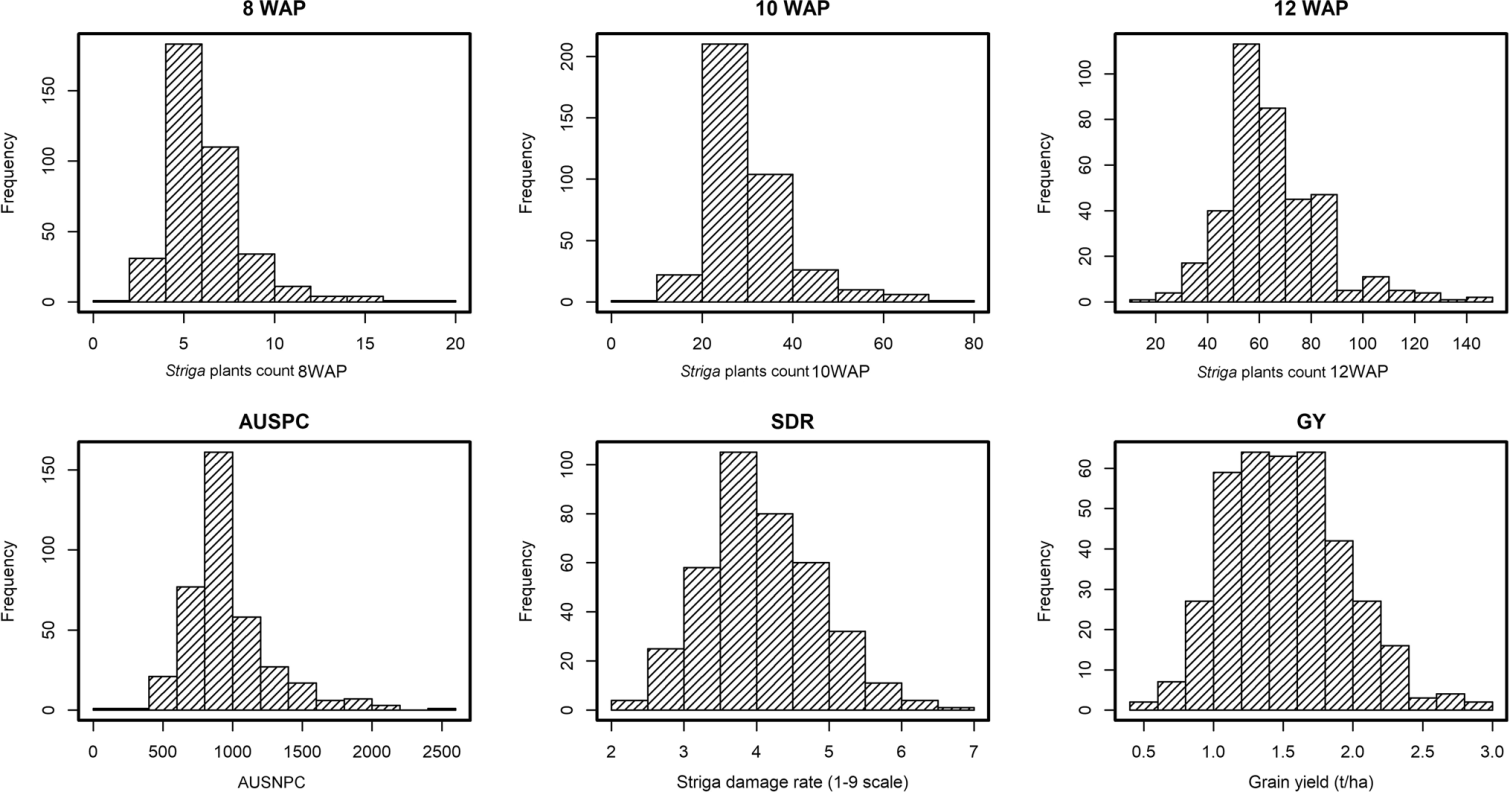
Phenotypic distribution of *Striga* resistance-related traits in the IMAS association mapping panel

Severity of *Striga hermonthica* infestation was moderate to high at the phenotyping locations. The average *Striga* plant count at 8 WAP was 6.2 which gradually increased to 30 and 65 after 10 and 12 WAP, respectively (Table 1). Location means for AUNSPC ranged from 189 to 2457 with the mean of 970. SDR scored 12 WAP ranged between 2 and 7. The GY of the 380 lines under artificial *Striga* infestation ranged from 0.0 to 3.5 tons ha^−1^, with an average of 1.52 tons ha^−1^. All measured traits showed significant (*P* < 0.05) genotypic and genotype-by-environment (G × E) interaction variances (Table 1). For traits associated with *Striga* resistance, the ratio of genotypic variance to G × E interaction variance was highest for SDR with 8.1 and lowest for *Striga* count at 8 WAP with 0.7. Heritability estimates under artificial *Striga* infestation were moderate (0.51–0.68) for *Striga* plant counts and AUSNPC, and high for SDR (0.84) and GY (0.70). The distribution of phenotypic values was unimodal for the number of emerged *Striga* plants at different intervals, AUSNPC and SDR indicating the quantitative nature of *Striga* resistance (Fig. 1).

**Table 1:** Means, ranges and components of variance for *Striga* hermonthica-related traits (under artificial infestation) for maize inbred lines in the IMAS association panel

Trait	Mean (range)	*σ*^2^_*G*_	*σ*^2^_*G* × *E*_	*σ*^2^_*e*_	*H*^*2*^
nStr_8WAP	6 (2–18)	10.13**	13.72**	31.24	0.51
nStr_10WAP	30 (9–79)	163.41**	159.44**	460.87	0.56
nStr_12WAP	65 (17–148)	579.53**	313.28**	990.88	0.68
AUSNPC	971 (190–2458)	1460.78**	1208.30**	2270.43	0.65
SDR	4.17 (2.18–6.54)	0.78**	0.10**	0.72	0.84
GY	1.52 (0.00-3.48)	0.28**	0.11**	0.53	0.70
AD	73 (62–84)	15.26**	1.45**	4.93	0.92
SD	76 (64–93)	20.58**	2.52**	8.53	0.90
PH	114.9 (83.8–162.8)	254.47**	7.98**	228.99	0.86
EH	54.4 (35.5–84.0)	98.00**	3.78**	96.93	0.85
EPP	1.1 (0.8–1.6)	0.02*	0.02*	0.14	0.42
HC	12.5 (2.3–99.9)	202.99**	56.90**	162.23	0.82
Pasp	3.4 (2.4–4.1)	0.11**	0.06*	0.21	0.66

nStr8WAP, nStr10WAP, nStr12WAP = *Striga* count at 8, 10 and 12 weeks after planting, respectively; *AUSNPC* Area under *Striga* number progress curve; *SDR Striga* damage rating at 12 weeks after planting in 1–9 scale; *GY* Grain yield under *Striga* artificial infestation in tons/ha; *AD* Days to anthesis; *SD* Days to silking; *PH* Plant height in cm; *EH* Ear height in cm; *EPP* Number of ears per plant; *HC* Husk cover in %; Pasp = Plant aspect in 1–5 scale

*and *significance at *P* < 0.05 and *P* < 0.01, respectively

**Fig. 2:**
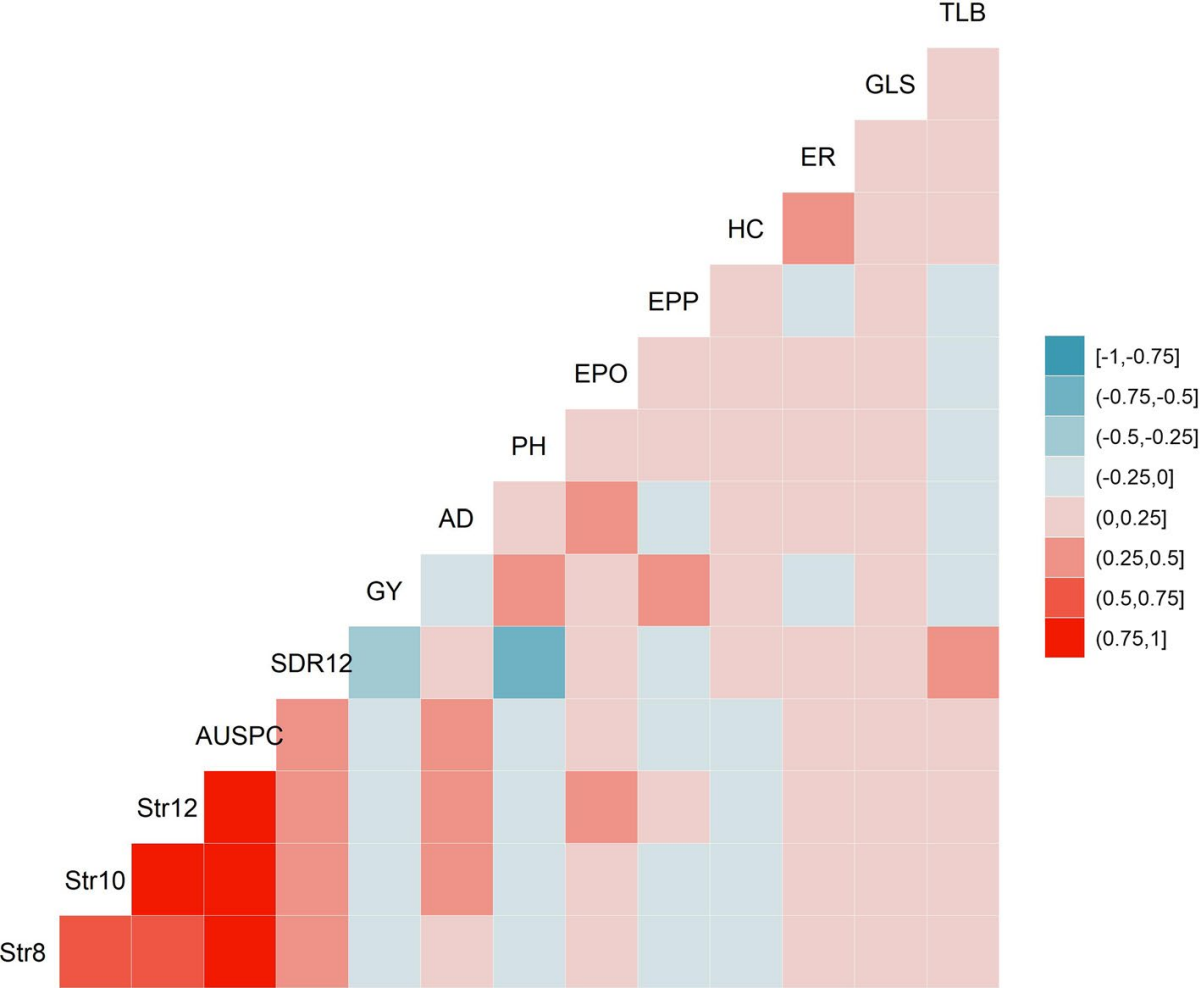
Correlations among fourteen measured traits. The correlation level is color-coded according to the color key plotted on the extreme right. Correlation values with > 0.12 and > 0.16 were significant at *P* = 0.05 and *P* = 0.01 levels, respectively. Str8, Str10, Str12 = *Striga* emerged plant count at 8, 10 and 12 weeks after planting, respectively; AUSPC = Area under *Striga* number progress curve; SDR12 = *Striga* damage rating at 12 weeks after planting; GY = Grain yield; AD = days to anthesis; PH = plant height, EPO = ear position; EPP = number of ears per plant; HC = husk cover; GLS = gray leaf spot; TLB = Turcicum leaf blight; ER = Fusarium ear rot

The strongest positive and significant correlations were observed between number of emerged *Striga* plants at 8, 10 and 12 WAP and AUNSPC (Fig. 2). SDR was positively and significantly correlated with number of emerged *Striga* plants at different intervals and AUSNPC. GY was significantly but negatively correlated with number of emerged *Striga* plants at different time intervals, AUSNPC and SDR. AD was positively correlated with number of emerged *Striga* plants at different time intervals, AUSNPC and SDR, and was negatively correlated with GY. HC was also negatively correlated with all *Striga* resistance indicator traits except for SDR. We observed significant correlations but of low magnitude between *Striga* resistance indicator traits and other agronomic traits. The correlation between GY and AUSNPC, as well as between GY and SDR, revealed many lines with good levels of resistance based on AUSNPC and SDR yielding > 2 tons per hectare of grain (Supplementary Figure S1).

The top 16 lines in terms of GY and their performance for other traits under *Striga* infestation are shown in Table 2. IMAS panel lines IMAS175 (DTPYC9-F46-1–2-1–2-B) and IMAS275 ((JL16.R119W)-1–1-#) had the highest GY coupled with low SDR. However, both IMAS175 (DTPYC9-F46-1–2-1–2-B) and IMAS275 ((JL16.R119W)-1–1-#) also supported a high number of *Striga* plants at 10 and 12 WAP. Inbred lines with high GY and supporting a low number of emerged *Striga* plants in addition to lower SDR were considered as resistant/tolerant to *Striga*.

**Table 2:** Performance of the best maize inbred lines for grain yield, number of *Striga* plants, *Striga* damage rating and agronomic traits under artificial *Striga* infestation across two years (2013 and 2014)

Genotype	Pedigree	GY (t/ha)	Number of *Striga* plants				SDR	AD	Additional information
			8 WAP	10 WAP	12 WAP	AUSNPC	12 WAP		
IMAS_175	DTPYC9-F46-1–2-1–2-B	3.48	3.4	19.6	26.4	507.9	2.6	65.8	Drought tolerant
IMAS_275	(JL16.R119W)-1–1-#	3.45	3.0	16.5	30.5	438.6	4.2	73.6	White, ARC South Africa
IMAS_131	CPHYS104	3.38	5.9	17.7	54.1	670.7	3.9	69.0	Drought sensitive
IMAS_241	CML454	3.37	4.8	13.8	42.7	456.1	4.3	76.8	Orange, lowland tropics
IMAS_255	R118W\R119W:(P4.VHKW)-4 U2540W)	3.25	4.7	26.4	60.6	842.6	3.5	71.6	White, ARC South Africa
IMAS_139	CPHYS112	3.25	4.7	24.4	55.1	775.9	3.5	68.4	Drought sensitive
IMAS_158	DTPWC9-F115-1–2-1–2-B	3.18	5.8	27.5	60.0	897.1	3.9	72.2	Drought tolerant
IMAS_346	Kit A1-3	3.08	8.2	28.9	51.9	900.6	2.9	69.5	White, Highlands, Kenya
IMAS_405	KIL-1	3.00	5.9	30.7	66.1	984.2	3.9	71.5	White, Mid-altitude, Kenya
IMAS_164	DTPWC9-F55-1–1-1–1-B	2.95	3.8	28.9	49.2	795.9	3.3	73.2	Low N tolerant
IMAS_261	[(TSELS(1)\15.(B2P36)S3\19]S 3.I137TNW)-1–1-1.U2540W)	2.84	6.3	27.7	64.4	935.1	3.7	69.2	White, ARC South Africa
IMAS_20	INTA/INTB-B-52-B-1–1-B	2.83	5.8	29.2	66.0	966.0	2.9	72.7	Low N tolerant
IMAS_312	CNO8Y/523	2.80	6.4	29.2	78.6	1047.2	3.5	72.4	Yellow, ARC South Africa
IMAS_117	CLQRCWQ108	2.77	6.2	39.3	65.0	1136.4	4.1	76.0	White, lowland tropics
IMAS_17	INTA/INTB-B-41-B-1–1-B	2.73	4.0	23.3	60.4	780.7	3.5	73.4	Low N sensitive
IMAS_309	CNO8Y/500	2.71	3.6	22.3	50.4	687.3	3.7	70.9	Yellow, ARC South Africa

8WAP, 10WAP, 12WAP = number of emerged *Striga* plants at 8, 10 and 12 weeks after planting, respectively; *AUSNPC* Area under *Striga* number progress curve; *SDR Striga* damage rating; *GY* Grain yield under artificial *Striga* infestation; *AD* Days to anthesis

**Fig. 3:**
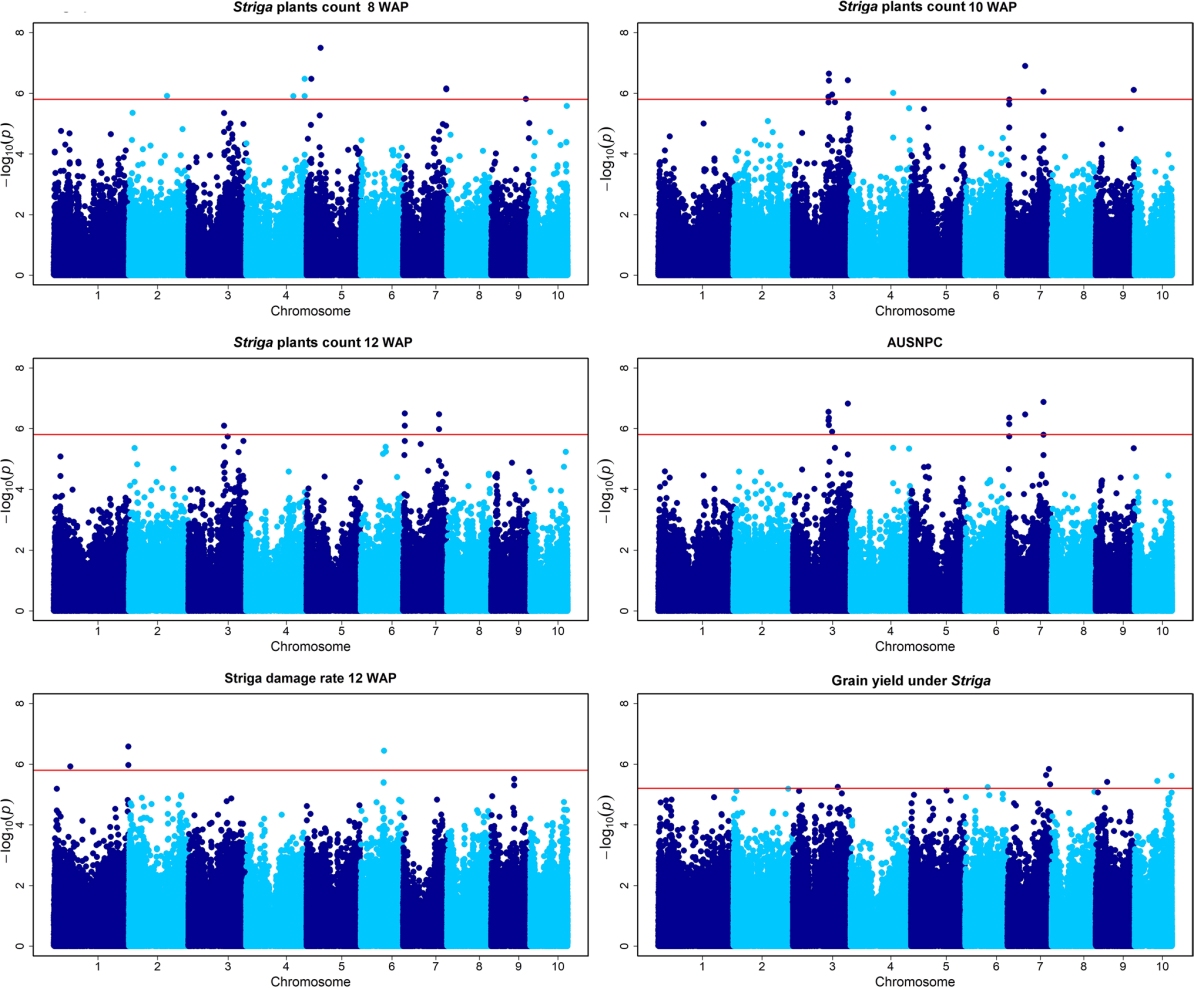
Manhattan plots of the GWAS for six different *Striga*-related traits in IMAS association mapping panel. The dashed horizontal line depicts the significance threshold (*P* = 2 × 10^–6^ for *Striga* resistance traits and *P* = 5.6 × 10^–6^ for GY). The *X*-axis indicates the SNP location along the 10 chromosomes, with chromosomes separated by different colors; *Y*-axis is the—log10(*P* observed) for each analysis

GWAS was performed for the number of emerged *Striga* plants at 8, 10 and 12 WAP, AUSNPC, SDR and GY. The results for the six traits are shown in Manhattan and Q–Q plots of *P* values comparing the expected *-log*_*10*_*p* values to observed  *− log*_*10*_*p* values (Fig. 3 and Supplementary Figure S3). GWAS detected 57 SNPs distributed across the genome that were significantly associated with the six different traits (Table 3; *P* = 2 × 10^–6^). Eleven, fourteen and six significant SNPs individually explained 8–10%, 8–11% and 8–10% of the total phenotypic variance for number of emerged *Striga* plants at 8, 10 and 12 WAP, respectively. For AUSNPC, SDR and GY, sets of 11, four and nine significant SNPs individually explaining 7–10%, 6–7% and 7–8% of the total phenotypic variance, respectively, were detected. The most significant SNP across the six traits was *S5_56842787* on chromosome 5 which explained 10% of the total phenotypic variance for number of emerged *Striga* plants at 8 WAP. SNPs *S5_56842787* on chromosome 5, *S7_70368510, S7_7160192* and *S7_144538472* on chromosome 7, *S1_298988628* on chromosome 1 and *S7_165944748* on chromosome 7 were found to be the most significantly associated for *Striga* count at 8, 10 and 12 WAP, AUSNPC, SDR and GY under *Striga*, respectively. A set of putative candidate genes associated with the significant markers was identified (Table 3). Additionally, the genome-wide linkage disequilibrium (LD) decay was plotted as LD (*r*^2^) between adjacent pair of markers and distance in kb (Figure S2). The average physical distance was 6.53 kb and 18.82 kb at a cut-off value of *r*^2^ = 0.2 and 0.1, respectively.

**Table 3:** Chromosomal positions and SNPs significantly associated with various *Striga* resistance-related traits detected by SNP-based GWAS in the IMAS association mapping panel

SNP-Name^a^	Chr	Position (bp)	MLM-P values	R^2^	MAF	Alleles	Putative candidate gene	Predicted function of candidate gene
*Number of Striga counts_8WAP*								
S2_152347470	2	152,347,470	1.23E-06	0.09	0.09	*G/*A	GRMZM2G314215	Unknown
S4_188749704	4	188,749,704	1.24E-06	0.09	0.11	*A/*G	GRMZM2G127230	Chloroplast signal recognition particle 54 kDa subunit (CPSRP54)
S4_234419757	4	234,419,757	3.35E-07	0.09	0.15	*T/*C	GRMZM2G146034	ABC transporters and multidrug resistance systems ABC transporter family
S4_234499448	4	234,499,448	1.25E-06	0.08	0.27	*A/*G	GRMZM2G145962	Ribosomal protein S21 family protein
S5_19133910	5	19,133,910	3.36E-07	0.09	0.07	*T/*C	GRMZM2G074871	Oxidoreductase activity, acting on paired donors, with incorporation or reduction of molecular oxygen
S5_56842787	5	56,842,787	3.20E-08	0.10	0.06	*G/*A	GRMZM2G077208	Protein synthesis, ribosomal protein, eukaryotic
S7_172979397	7	172,979,397	6.95E-07	0.08	0.05	*A/*G	GRMZM2G053554	Minor CHO metabolism, galactose, alpha-galactosidases Melibiase family protein
S7_172979399	7	172,979,399	6.95E-07	0.08	0.05	*A/*C		
S7_172979402	7	172,979,402	6.95E-07	0.08	0.05	*A/*C		
S7_172979403	7	172,979,403	7.44E-07	0.09	0.06	*C/*G		
S9_139760291	9	139,760,291	1.55E-06	0.08	0.05	*T/*C	GRMZM2G114126	Cell vesicle transport OSBP (oxysterol binding protein)-related protein 3C (ORP3C)
*Number of Striga counts_10WAP*								
S3_143803575	3	143,803,575	1.31E-06	0.08	0.05	*T/*C	GRMZM2G164502	Signaling, receptor kinases, LRR I LRR protein kinase family protein
S3_143804650	3	143,804,650	1.99E-06	0.09	0.06	*C/*G		
S3_145603175	3	145,603,175	2.26E-07	0.08	0.07	*T/*C	GRMZM2G061206	Proteinaceous RNase P 2
S3_145603187	3	145,603,187	3.87E-07	0.08	0.06	*A/*G		
S3_158421414	3	158,421,414	1.11E-06	0.09	0.05	*A/*G	GRMZM2G092112	Protein degradation, autophagy encodes autophagy protein 6 (ATG6), required for pollen germination and plant development
S3_169078369	3	169,078,369	1.97E-06	0.08	0.06	*A/*C	GRMZM2G026855	Alcohol dehydrogenases zinc-binding dehydrogenase family protein
S3_169078370	3	169,078,370	1.97E-06	0.08	0.06	*T/*C		
S3_169078372	3	169,078,372	1.97E-06	0.08	0.06	*T/*C		
S3_220734197	3	220,734,197	3.74E-07	0.08	0.08	*A/*C	GRMZM2G121313	Unknown
S4_170790739	4	170,790,739	9.71E-07	0.08	0.06	*G/*A	GRMZM2G075368	Hormone metabolism, ethylene signal transduction involved in ethylene perception in Arabidopsis ethylene response 2 (ETR2)
S7_7162177	7	7,162,177	1.62E-06	0.08	0.08	*A/*C	GRMZM2G018508	Protein degradation, ubiquitin ubiquitin-specific protease 13 (UBP13)
S7_70368510	7	70,368,510	1.27E-07	0.11	0.05	*T/*C	GRMZM5G892471	Unknown
S7_144538472	7	144,538,472	8.71E-07	0.09	0.42	*T/*C	GRMZM2G015520	Basic helix-loop-helix (bHLH) DNA-binding superfamily protein
S9_153482728	9	153,482,728	7.80E-07	0.08	0.05	*C/*T	GRMZM2G171986	Unknown
*Number of Striga counts_12WAP*								
S3_143804650	3	143,804,650	8.08E-07	0.10	0.06	*G/*C	GRMZM2G164502	Signaling, receptor kinases, LRR I LRR protein kinase family protein
S3_158421414	3	158,421,414	1.83E-06	0.09	0.05	*A/*G	GRMZM2G092112	Protein degradation, autophagy encodes autophagy protein 6 (ATG6), required for pollen germination and plant development
S7_7160182	7	7,160,182	8.05E-07	0.08	0.08	*T/*A	GRMZM2G018508	Protein degradation. ubiquitin ubiquitin-specific protease 13
S7_7160192	7	7,160,192	3.19E-07	0.09	0.08	*T/*A		
S7_144404387	7	144,404,387	1.05E-06	0.08	0.05	*G/*C	GRMZM2G450069	Unknown
S7_144538472	7	144,538,472	3.36E-07	0.09	0.42	*T/*C	GRMZM2G015520	Basic helix-loop-helix (bHLH) DNA-binding superfamily protein
*Area under number of Striga counts progress curve (AUNSPC)*								
S3_143803575	3	143,803,575	5.37E-07	0.08	0.05	*T/*C	GRMZM2G164502	Signaling, receptor kinases, LRR I LRR protein kinase family protein
S3_143804650	3	143,804,650	2.79E-07	0.10	0.06	*G/*C		
S3_145603175	3	145,603,175	4.41E-07	0.08	0.07	*T/*C	GRMZM2G061206	Proteinaceous RNase P 1
S3_145603187	3	145,603,187	7.74E-07	0.08	0.06	*A/*G		
S3_158421414	3	158,421,414	1.28E-06	0.09	0.05	*A/*G	GRMZM2G092112	Protein degradation, autophagy encodes autophagy protein 6 (ATG6), required for pollen germination and plant development
S3_220734197	3	220,734,197	1.49E-07	0.09	0.08	*A/*C	GRMZM2G121313	Unknown
S7_7160182	7	7,160,182	1.80E-06	0.08	0.08	*T/*A	GRMZM2G018508	Protein degradation, ubiquitin ubiquitin-specific protease 13 (UBP13)
S7_7160192	7	7,160,192	4.40E-07	0.08	0.08	*T/*A		
S7_7162177	7	7,162,177	7.17E-07	0.09	0.08	*A/*C		
S7_70368510	7	70,368,510	3.41E-07	0.10	0.05	*T/*C	GRMZM5G892471	Unknown
S7_144404387	7	144,404,387	1.59E-06	0.07	0.05	*G/*C	GRMZM2G450069	Unknown protein
S7_144538472	7	144,538,472	1.33E-07	0.10	0.42	*T/*C	GRMZM2G015520	Basic helix-loop-helix (bHLH) DNA-binding superfamily protein
*Striga damage rate_12WAP*								
S1_66175831	1	66,175,831	1.19E-06	0.09	0.46	*C/*T	GRMZM2G143086	Uncharacterized protein
S1_298964314	1	298,964,314	1.07E-06	0.10	0.06	*T/*G	GRMZM2G422670	Lipid metabolism, lipid degradation, lysophospholipases phosphoinositide phospholipase
S1_298988628	1	298,988,628	2.61E-07	0.09	0.08	*G/*A	GRMZM2G099987	Ribonuclease P protein subunit P38-related
S6_93323528	6	93,323,528	3.58E-07	0.09	0.25	*A/*G	GRMZM2G143782	Protein.degradation.ubiquitin.E3.BTB/POZ Cullin3.BTB/POZ BTB-POZ and MATH domain 1 (BPM1)
*Grain yield under Striga infestation*								
S3_180966178	3	180,966,178	5.65E-06	0.07	0.05	*A/*G	GRMZM2G094771	O-fucosyltransferase family protein
S6_89412913	6	89,412,913	5.69E-06	0.07	0.22	*C/*G	GRMZM2G023051	uncharacterized protein
S7_154508166	7	154,508,166	2.29E-06	0.08	0.18	*T/*C	GRMZM2G468657	Protein degradation, aspartate protease Eukaryotic aspartyl protease family
S7_165944748	7	165,944,748	1.44E-06	0.08	0.18	*C/*A	GRMZM2G086856	Uncharacterized protein
S7_165944751	7	165,944,751	1.44E-06	0.08	0.18	*C/*G		
S7_171043455	7	171,043,455	4.62E-06	0.07	0.07	*A/*G	GRMZM2G326263	Pentatricopeptide (PPR) repeat-containing protein
S7_171043478	7	171,043,478	4.62E-06	0.07	0.07	*A/*G		
S9_46386574	9	46,386,574	3.82E-06	0.07	0.07	*G/*T	GRMZM2G115329	Uncharacterized protein
S10_91087034	10	91,087,034	3.57E-06	0.08	0.08	*T/*C	GRMZM5G876837	Unknown
S10_148086732	10	148,086,732	2.44E-06	0.08	0.11	*A/*G	GRMZM2G343144	Gluco-, galacto- and mannosidases. endoglucanase glycosyl hydrolase 9C1

Chr = chromosome; *MAF* Minor Allele Frequency; Alleles italic represent minor alleles; *P* value is for mixed linear model;

^a^The exact physical position of the SNP can be inferred from marker’s name, for example, S1_82702920: chromosome 1; 82,702,920 bp

**Fig. 4:**
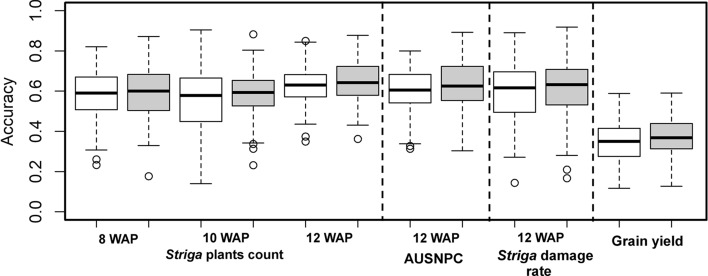
Distribution of the accuracy of genomic predictions for five different *Striga* related traits and grain yield under artificial *Striga* infestation. Prediction was based on random markers (white box) as well as combined prediction based on random markers and trait-associated markers (gray box) detected through GWAS with fivefold cross-validations in IMAS association panel of 380 inbred lines

GP was applied to evaluate the accuracy with different cross-validation methods with individual locations as well as across locations. Average correlations between predictions and observed phenotypes in CV0, CV1 and CV2 for all the measured traits with and without G × E interaction effects are presented in Table 4 and Fig. 4. Among the three different cross-validations, CV1 performed poorly compared with CV0 and CV2 for measured traits, with CV0 and CV2 performing equally well. Prediction for one environment (Alupe 2014) showed better correlations for number of emerged *Striga* plants at 8, 10 and 12 WAP and AUSNPC compared with the other environments, whereas for SDR and GY, Alupe2013 performed better. There were slight increases in the prediction correlations for models with the G × E interaction in cross-validation scenarios for most traits and environments. Prediction correlations were also obtained for the measured traits using BLUEs across locations which showed values similar to those observed for CV0 and CV2 (Fig. 4). Inclusion of significant markers detected through GWAS in the prediction model showed slight increase in prediction correlations for all the *Striga* resistance indicator traits and GY. The prediction correlations were higher for all *Striga* resistance-related traits compared with GY. For GY in CV2, we observed a similar increase in the prediction correlations for two out of three environments. The two environments Alupe 2014 and Kibos 2013 had prediction correlations of 0.376 and 0.505 for a model without G × E and 0.411 and 0.518 for a model with G × E, respectively.

**Table 4:** Genomic prediction accuracies for *Striga* resistance-related traits, including grain yield under artificial *Striga* infestation

Trait	Environment	CV0		CV1		CV2	
		G	G + GE	G	G + GE	G	G + GE
Str8WAP	Alupe2013	0.377	0.401	0.234	0.226	0.390	0.404
	Alupe2014	0.508	*0.512*	*0.352*	0.342	0.461	*0.470*
	Kibos2013	0.347	0.339	0.260	0.248	0.341	0.331
Str10WAP	Alupe2013	0.461	0.483	0.281	0.265	0.468	*0.484*
	Alupe2014	*0.541*	0.537	*0.359*	0.343	0.468	*0.478*
	Kibos2013	0.305	0.309	0.213	0.203	0.313	0.313
Str12WAP	Alupe2013	0.521	0.542	0.386	0.363	0.550	0.559
	Alupe2014	0.616	*0.618*	*0.417*	0.404	0.546	*0.561*
	Kibos2013	0.483	0.492	0.312	0.294	0.473	0.485
AUSNPC	Alupe2013	0.515	0.543	0.346	0.326	0.540	0.557
	Alupe2014	0.625	*0.626*	*0.424*	0.407	0.547	*0.560*
	Kibos2013	0.409	0.416	0.266	0.252	0.406	0.413
SDR	Alupe2013	*0.668*	0.665	*0.425*	0.421	*0.665*	*0.665*
	Alupe2014	0.586	0.590	0.331	0.324	0.586	0.591
	Kibos2013	0.631	0.635	0.334	0.325	0.647	0.651
GY	Alupe2013	0.537	*0.541*	0.351	*0.348*	*0.551*	*0.551*
	Alupe2014	0.489	0.491	0.109	0.093	0.376	0.411
	Kibos2013	0.444	0.482	0.218	0.197	0.505	0.518

Average correlations from fivefold cross-validation between the predicted and observed values of genotypes for each and across environments for models with and without G × E effects with three different cross‐validation schemes (CV0, CV1 and CV2). For CV0, CV1 and CV2, the best predicted correlations are italicized

nStr8WAP, nStr10WAP, nStr12WAP = *Striga* plants at 8, 10 and 12 weeks after planting, respectively; *AUSNPC* Area under *Striga* number progress curve; *SDR Striga* damage rating at 12 weeks after planting; *GY* Grain yield under *Striga* infestation

We compared prediction accuracies from the GBLUP model with the IBCF approach with CV2 (Table 5). For IBCF, the training population included all the traits from any two environments while the prediction set included the target traits in the remaining third environment. GY was predicted with moderate accuracy with IBCF approach, with 0.010, and 0.013 increases in accuracy in location Alupe 2014 and Kibos 2013, respectively. However, in Alupe 2013, a decrease (-0.004) in accuracy compared with the GBLUP model was observed. For all *Striga* resistance indicator traits, the prediction accuracy was at least 15% higher with the IBCF approach compared with the GBLUP model in all locations (Table 5). Overall, with the IBCF approach, a maximum increase of 0.40 was observed for number of emerged *Striga* plants at 10 WAP and AUSNPC for Alupe2013 over the GBLUP model.

**Table 5:** Prediction correlations for lines with missing phenotypic data at specific environments: Alupe2013, Alupe2014 and Kibos2013 under cross-validation scheme CV2, using item-based collaborative filtering (IBCF) and genomic best linear unbiased prediction (GBLUP) methods

Trait	Testing environment							
	IBCF				GBLUP			
	Alupe 2013	Alupe 2014	Kibos 2013	Mean	Alupe 2013	Alupe 2014	Kibos 2013	Mean
GY	0.547	0.386	0.518	0.484	0.551	0.376	0.505	0.477
Str8WAP	0.747	0.732	0.568	0.682	0.390	0.461	0.341	0.398
Str10WAP	0.872	0.825	0.610	0.769	0.468	0.468	0.313	0.416
Str12WAP	0.827	0.794	0.638	0.753	0.550	0.546	0.473	0.523
AUSNPC	0.933	0.880	0.685	0.833	0.540	0.547	0.406	0.498
SDR	0.860	0.866	0.812	0.846	0.665	0.586	0.647	0.633
PH	0.798	0.642	0.732	0.724	0.703	0.616	0.694	0.671
EH	0.919	0.853	0.844	0.872	0.697	0.558	0.700	0.652
AD	0.799	0.545	0.677	0.674	0.823	0.784	0.819	0.809

Trait phenotypes in one environment were predicted using data from other two environments

nStr8WAP, nStr10WAP, nStr12WAP = number of emerged *Striga* plants at 8, 10 and 12 weeks after planting, respectively; *AUSNPC* Area under *Striga* number progress curve; *SDR Striga* damage rating at 12 weeks after planting; *GY* Grain yield under *Striga* infestation; *AD* Days to anthesis; *PH* Plant height; *EH* Ear height

## Discussion

The defense mechanisms of maize against *Striga* were grouped into resistance and tolerance (Kim 1994). The ability of the host plant to withstand the effects of the *Striga* plants that are already attached, regardless of their number was termed as tolerance. It is quantified by a host damage syndrome rating score using a 1–9 scale (Kim 1994). Ability of the host plant to prevent the parasite from attaching itself to the roots was referred to as resistance (Kim 1994). This is quantified by the number of emerged *Striga* plants around the base of the host plant. Resistance mechanisms were also further categorized as pre‐attachment and post‐attachment. Mechanisms that prevent or reduce *Striga* seed germination rates were categorized as pre‐attachment resistance, while those that prevent or reduce the success of root penetration or establishment of the vascular connection between host and parasite were called post‐attachment resistance (Yoder and Scholes 2010). Both host damage score and *Striga* emergence, along with GY under *Striga* infestation, were considered as the most appropriate criteria to use in breeding for *Striga* tolerance/resistance (Kim 1991; Kim et al. 1998; Badu-Apraku and Fakorede 1999; Badu-Apraku and Akinwale 2011).

Genetic variability is important in efficient selection for improved GY under stress environments such as *Striga*-infested conditions. The observed significant genotypic variance for the measured traits in the present study showed the potential for selection of improved GY under *Striga*-infested conditions. There was a wide range of responses to *Striga* infestation in terms of GY and *Striga* resistance parameters. The inbred lines used in this study were from diverse geographical regions with different breeding histories and this may have contributed to the variation observed. The lines showing good response to artificial *Striga* infestation should be utilized in more detailed studies to ascertain the resistance mechanisms involved. The significant G × E variances for the measured traits indicated that they are highly affected by the G × E interaction which could be attributed to variation in climatic and soil conditions across the two years. Previous studies have also reported significant G × E under *Striga*-infested conditions (Menkir et al. 2012; Makumbi et al. 2015; Kanampiu et al. 2018). Moderate to high heritability estimates were observed indicating the potential for these traits to be improved through recurrent selection. Broad-sense heritability is an estimate of the upper boundary of the narrow-sense heritability. High broad-sense heritability estimates for GY, AUSNPC, SDR and number of emerged *Striga* plants 12 WAP suggest that the actual narrow-sense heritability could be higher and that reasonable genetic gain for these traits could be expected. The high broad-sense heritability estimates for GY (0.70) and number of emerged *Striga* plants 12 WAP (0.68) observed in this study corroborate previous reports under artificial *Striga* infestation (Menkir et al. 2012; Makumbi et al. 2015; Adewale et al. 2020) and higher than the heritability observed in biparental populations (Badu-Apraku et al. 2020a, b).

The significant negative correlation between GY and number of emerged *Striga* plants indicated that increase in number of emerged *Striga* plants led to severe reduction in GY (Menkir et al. 2012; Adewale et al. 2020). For SDR, tolerance is associated with lower values in the 1–9 scale and thus significant negative correlation between SDR and GY implied that lower SDR values were associated with improved GY. The observed positive and significant correlations between number of emerged *Striga* plants, SDR and AUNSPC suggest that these traits can be combined into an index for selection under *Striga* infestation.

The detection power of GWAS depends on the LD between the QTL and the markers. Cross-pollinated crops like maize have more rapid LD decay compared with self-pollinated crops due to outcrossing and consequently high genetic diversity (Kaler et al. 2020). The results of the present study indicated that the LD decayed rapidly across the physical distance (18.82 kb and 6.53 kb at a cut-off value of *r*^2^ = 0.1 and 0.2; Supplementary Figure S2) indicating that the IMAS association mapping panel has significant diversity, mimicking a natural population, and thus was appropriate for conducting GWAS. Previous studies on the population structure of the IMAS panel used in this study showed confounding structure in the panel (Gowda et al. 2015; Ertiro et al. 2020).

Correcting for population structure is an important step in GWAS to reduce the false positives that could arise from it without overcorrecting and further causing false negatives (Jaiswal et al. 2019). Another factor that leads to false positives is the more recent common ancestry and family relatedness which is controlled by the inclusion of a kinship model through the identity by state approach (Loiselle et al. 1995). Therefore, we incorporated the population structure and kinship matrix as covariates (Q + K) in the mixed linear model. The Q–Q plots of the six traits showed proper distribution of the observed over the expected *P* values indicating that the model and the comparison method used were an appropriate fit in this GWAS approach (Supplementary Figure S3).

At a significant threshold *p* value (*p* = 2 × 10^–6^ for *Striga* resistance traits and *P* = 5.6 × 10^–6^ for GY), we identified a total of 57 marker trait associations distributed across the genome and controlled by few major and many minor effect QTL (6–11% of the total phenotypic variance) suggesting the complexity of resistance to *Striga* infestation. For SDR and number of emerged *Striga* plants at 8 WAP, we observed four and 11 SNPs and Adewale et al (2020) reported nine and one significantly associated SNPs, respectively. However, no overlapping SNPs were observed across the studies for both the traits possibly due to different timing of data scored and the different materials used in the study. On the other hand, one SNP *S3_158421414* detected consistently for number of emerged *Striga* plants at 10 and 12 WAP and AUSNPC was overlapped with the QTL reported by Badu-Apraku et al (2020a) in biparental population. In this study, no overlapping of SNPs was detected between number of *Striga*-emerged plants and SDR, however five SNPs on chromosome 3 (*S3_143803575, S3_143804650, S3_145603175, S3_145603187* and *S3_158421414*) detected for number of *Striga*-emerged plants were overlapped with four QTL detected for SDR in an earlier study (Badu-Apraku et al 2020b) which suggests this region might be carrying an important gene/s for resistance to *Striga*.

A set of putative candidate genes associated with the significant markers was identified; their functions indicated their direct or indirect involvement in plant defense responses (Table 3). These candidate genes may be useful after validation in breeding for *Striga* resistance through marker-assisted selection. Out of the nine, two candidate genes: *GRMZM2G018508* and *GRMZM2G015520* were most significantly associated with number of emerged *Striga* plants at 10 and 12 WAP, respectively. *GRMZM2G018508* was identified to be involved in the ubiquitination processes (Zhou et al. 2017), while *GRMZM2G015520* is involved in signal- and stress-related regulated pathways of the transcription factors (Niu et al. 2017) that play important roles in plant responses to stress.

The other seven candidate genes are involved in plant responses to stress through various mechanisms ranging from metabolism and biosynthesis of compounds to detoxification processes. In particular, *GRMZM2G127230* is linked to chloroplast recognition particle (cpSRP) involved in the post-translational targeting of the nuclear encoding light harvesting chlorophyll-binding proteins (LHCPs) to the thylakoid membrane (Funke et al. 2005). The LHCPs members are positively involved in the abscisic acid (ABA) signaling in the stomata movement and plant responses to stress (Funke et al. 2005). *GRMZM2G146034* is linked to the ABC transporters found in the plant cell membranes and is involved in the detoxification processes, response to abiotic stresses, pathogen resistance and interaction of the plant with its environment (Choi 2005). *GRMZM2G074871* was found to be involved in the metabolism of a wide variety of exogenous and endogenous compounds, biosynthesis of pigments, volatiles, antioxidants, allelochemicals and defense compounds including phenolics and conjugates through the heme–thiolate proteins in specific, the cytochrome P450s (Chadha et al. 2018).

Two candidate genes were found to be involved in the degradation processes important in plant defense. *GRMZM2G092112* is involved in protein degradation and autophagy processes that allow the plant to perceive and react to invading pathogens and thus are involved in plant immunity responses through the regulation of programmed cell death (Fujiki et al. 2007). Another candidate gene, *GRMZM2G075368,* is involved in the degradation of ethylene receptors and signal transduction (Chen et al. 2007). The production of ethylene is tightly regulated by internal stimuli during development and in response to environmental stimuli from biotic and abiotic stresses (Chen et al. 2007). The other candidate gene *GRMZM2G164502* plays a role in plant stress responses through Leucine-Rich Repeats Receptor-Like Kinase which acts as mediators of cell-to-cell communication to relay environmental stimuli or to activate defense/resistance pathways against pathogens (Dufayard et al. 2017). Further studies on the candidate genes can pave the way for their potential use in breeding for *Striga* resistance.

GP has been widely applied in plant breeding to circumvent the drawbacks of marker-assisted selection by capturing all marker effects. GP that includes G × E interaction into the model substantially improves the accuracy (Guo et al. 2013). However, the presence of G × E negatively affects the heritability of traits thereby limiting the selection process (Roorkiwal et al. 2018). The assessment of GP correlations for measured traits with and without G × E using the three CVs revealed that CV1 performed relatively poor as compared to CV0 and CV2. The lower prediction correlations shown in CV1 corroborate the results from previous studies (Burgueño et al. 2012; Jarquín et al. 2014). Overall, CV1 scenario is more challenging than CV2, as in CV1, we are trying to predict the performance of newly developed lines (not tested in the field), whereas in CV2, we predict the performance of lines that have not been evaluated in some environments but which have been evaluated in different correlated environments. This is reflected in the results that showed CV‐correlations obtained in CV2 to be 12, 13, 15, 15, 27 and 25% greater than those obtained in CV1 for number of emerged *Striga* plants at 8WAP, 10WAP, 12WAP, AUSNPC, SDR and GY, respectively. This emphasizes the importance of having information from correlated environments when predicting performance. Selection of lines without field testing, as mimicked in CV1, allows the reduction of the breeding cycle interval, but the lower prediction accuracy might negatively affect the rate of genetic gain in a breeding program. Ultimately, the trade-off between desired prediction accuracy and generation interval depends on the structure of the breeding scheme.

For *Striga* resistance indicator traits, we observed an increase of up to 4% in the prediction accuracy with CV0 and CV2 but not much change was observed under CV1 after incorporating the G × E interaction in the model. Similar to observed prediction correlations for GY in CV2, Burgueño et al. (2012) and Jarquín et al. (2014) also reported prediction correlations of 0.439 and 0.475 (for a model without G × E) and of 0.556 and 0.514 (for a model with G × E), respectively, for GY in wheat. These results show the importance of incorporating the G × E interaction in the model. The observed slight differences in the prediction correlations between the studies are possibly due to model differences, the trait under study and the heritability. Overall, the prediction ability obtained in this study with CV0 and CV2, as well as observed accuracy based on BLUEs across locations, is high enough to warrant implementation of GP in practical breeding for *Striga* resistance in maize. The prediction accuracies based on BLUEs across locations, with and without inclusion of GWAS-detected markers, are moderately high. This is slightly overestimated since we fitted the markers detected with whole population-based GWAS rather training population alone. Nevertheless, we observed very small change in prediction accuracy by including GWAS-detected markers into the prediction model which supports *Striga* resistance indicator traits are governed by more of small to moderate effect genes/QTLs rather by major effect genes.

The IBCF approach was used to predict *Striga* resistance indicator traits and GY one at a time by integrating information from multiple traits evaluated in correlated environments. We observed an increase in accuracy for GY and *Striga* resistance indicator traits over the GBLUP model for all three locations. On the other hand, the GBLUP model outperformed the IBCF approach for all three locations for AD. The moderately low correlations obtained with the IBCF approach for GY compared with high prediction correlations for *Striga* resistance indicator traits might be due to the low magnitude of correlation of other traits with GY, as compared to each of the *Striga*-related traits. This indicates that changes in correlations of target trait with related traits will affect the predictive ability, especially when there is low correlation between selected locations and traits. While the ability of the IBCF approach resulted correlations higher than the GP correlations specifically for all *Striga* resistance indicator traits supports the utility of the method for improving *Striga* resistance breeding. This is also clearly supported by observed high correlations between *Striga* resistance indicator traits like number of emerged *Striga* plants at 8WAP, 10WAP, 12WAP and AUSNPC (Fig. 2). The observed prediction correlations are based on single-trait model and it is possible to improve further with multiple trait-based models. In rice for water usage trait data collected at different time intervals, multiple-trait regression models showed better prediction accuracy over single-trait regression model (Baba et al. 2020). Alternatively, from the perspective of traditional multiple-trait selection, we can also use the phenotype value of the closest related trait as the predicted value of the target trait. Overall, IBCF approach is very useful when the number of traits or environments, as well as correlations between them, is large. Moreover, its implementation is very fast as it can be used on very large data sets with limited computational power.

## Conclusion

In this study, we evaluated an association mapping panel of 380 inbred lines in multiple locations in Western Kenya under artificial *S. hermonthica* infestation to understand the genetic architecture of traits related to resistance to *Striga.* GWAS results revealed the polygenic nature of *Striga* resistance indicator traits with moderate genetic effects and significant G × E. We demonstrated that GWAS together with GP could potentially increase the efficiency of breeding for *Striga* resistance by improving the prediction accuracy. We found a significant improvement in prediction performance of *Striga* resistance indicator traits when G $\times$ E interaction was taken into account under the CV0 and CV2 cross-validation approaches where prediction is for the line’s performance in new environments. Results suggested that integration of GP in maize breeding even with diverse germplasm like this IMAS association mapping panel could effectively complement phenotypic selection to improve resistance to *Striga*, besides significantly reducing time and cost of breeding. Incorporation of the IBCF approach could further improve selection decisions in breeding for *Striga* resistance.

## Supplementary information

Below is the link to the electronic supplementary material.

Supplementary file1 (DOCX 408 kb)

## Data Availability

All datasets generated for this study are included in the article/Supplementary material.
