# Supplementary material for: Genetic dissection of Striga hermonthica (Del.) Benth. resistance via genome-wide association and genomic prediction in tropical maize germplasm
Source: Theor Appl Genet. 2021 Jan 3;134(3):941–58. doi: 10.1007/s00122-020-03744-4 (PMC7925482; doi:10.1007/s00122-020-03744-4)
Supplement: Supplementary file 1 — Supplementary file1 (DOCX 408 kb) [file 122_2020_3744_MOESM1_ESM.docx]

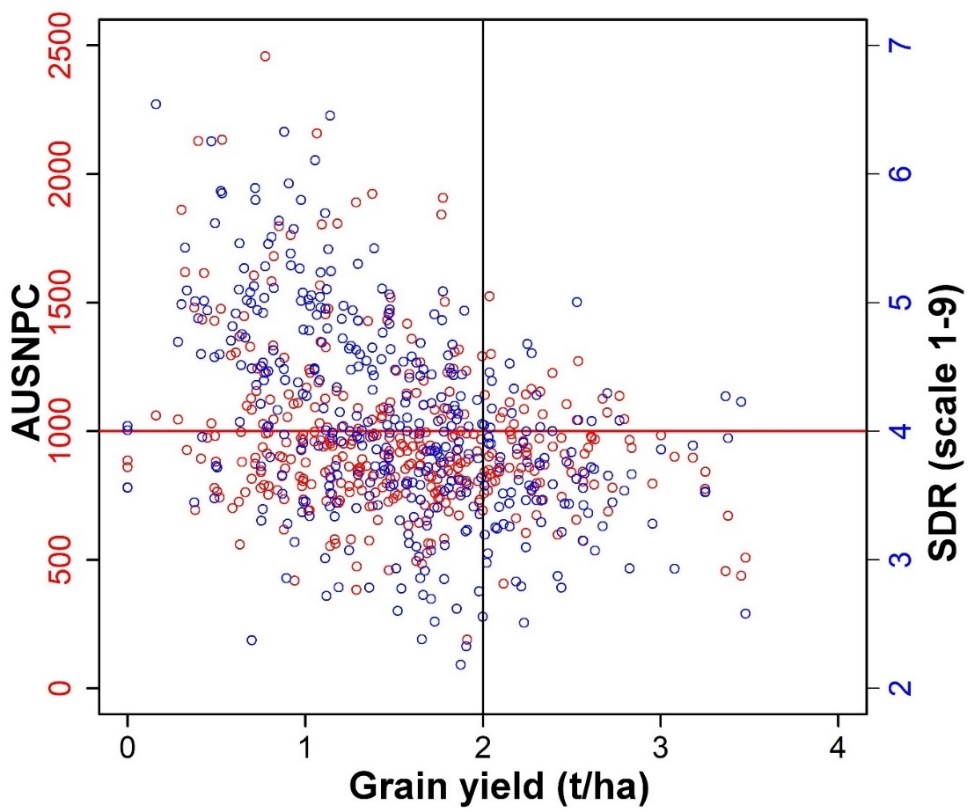


**Supplementary Figure S1.** Correlation of grain yield with area under Striga number progress curve (AUSNPC, in red circle) and Striga damage rating (SDR in blue circle).


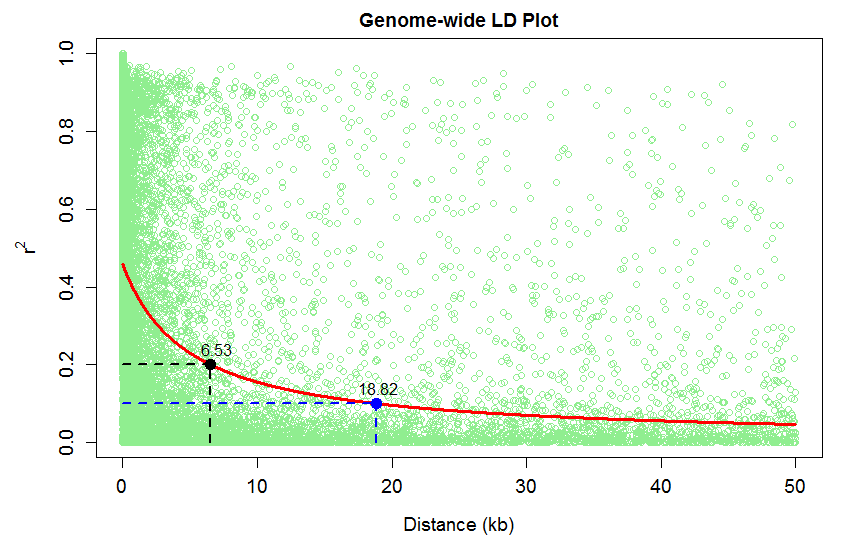


**Supplementary Figure S2.** Linkage disequilibrium (LD) plot representing the average genome-wide LD decay in the IMAS panel. Y axis represents the squared correlation coefficient r^2^ while the X axis represents the physical distance on the chromosome.


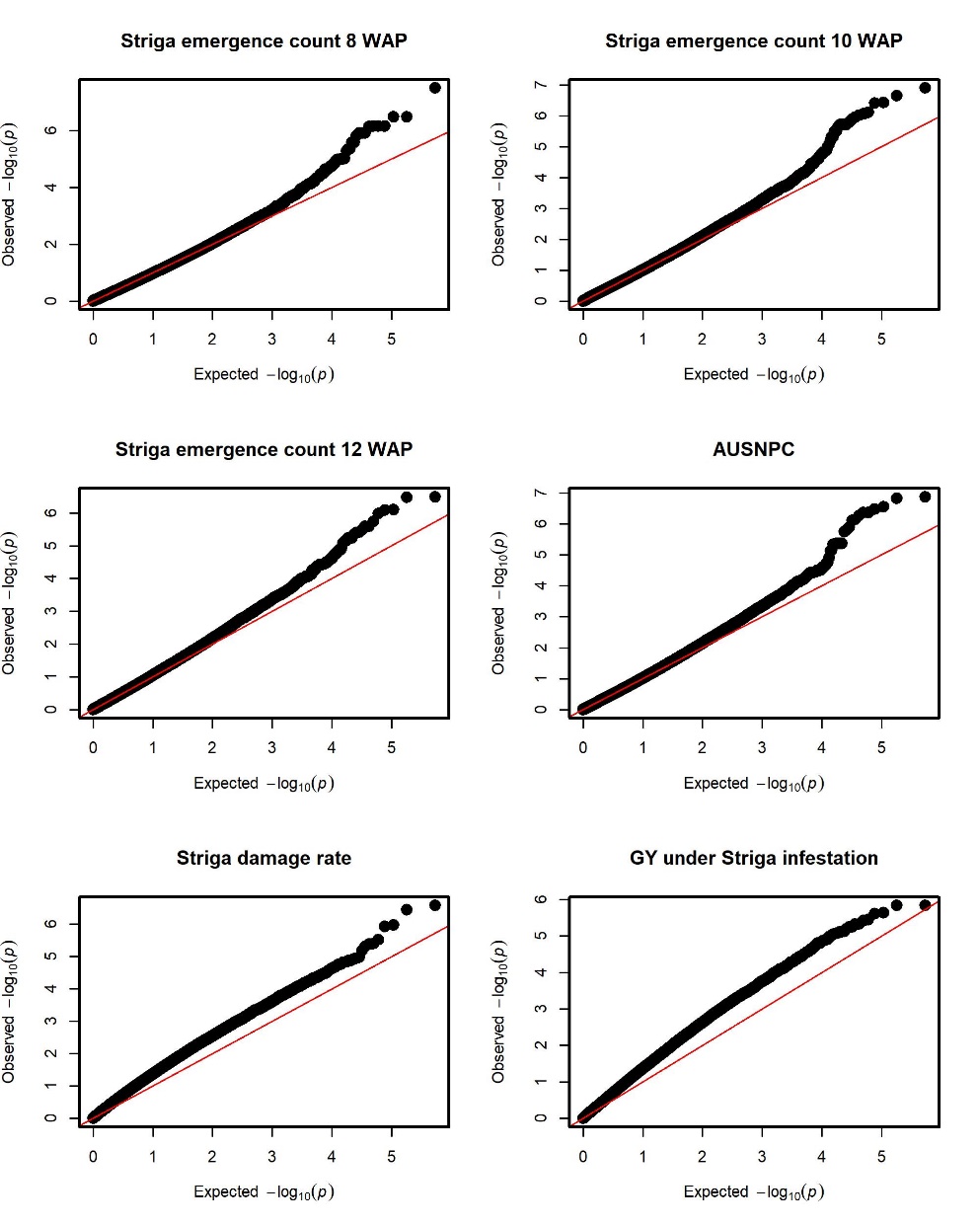


**Supplementary Figure S3**. Quantile-quantile plots for *Striga* *hermonthica* resistance related traits in the IMAS association panel.
